# Supplementary material for: Variation in prevalence of participation limitation, injury location, and training habits between young and master runners: A cross-sectional study
Source: PLoS One. 2026 May 5;21(5):e0346973. doi: 10.1371/journal.pone.0346973 (PMC13143076; doi:10.1371/journal.pone.0346973)
Supplement: Attachment — Submitted filename: PONE-D-25-60207_reviewer_reviewer comments.pdf [file pone.0346973.s001.pdf]

PLOS ONE

Variation in prevalence of participation limitation, injury location, and training habits between young and master runners: a cross-sectional study

--Manuscript Draft--

**Manuscript Number:**	PONE-D-25-60207
**Article Type:**	Research Article
**Full Title:**	Variation in prevalence of participation limitation, injury location, and training habits between young and master runners: a cross-sectional study
**Short Title:**	Running injury in master runners
**Corresponding Author:**	Rachel Berns<br>University of Southern California<br>Los Angeles, UNITED STATES OF AMERICA
**Keywords:**	age-related differences; overuse; running injuries; training characteristics
**Abstract:**	Background/Objectives: Running is a popular form of physical activity, but its growing participation has been accompanied by high rates of injury that may limit ongoing engagement. This study explored differences in running participation limitation, injury location, and training characteristics between young (18–34 years) and master (>35 years) runners. Methods: A total of 207 runners (105 young, 102 master) completed an online survey assessing demographics, training habits, and injury-related participation limitations using the Oslo Sports Trauma Research Center Overuse Injury Questionnaire. Results: Master runners reported significantly higher rates of injury-related participation limitation than young runners (51% vs. 34%, p<0.05) and more upper extremity injuries (p<0.01). They also had longer running histories, higher weekly mileage, and longer training durations (p<0.05). Among master runners, risk factors for reduced participation included running on gravel/pebbled surfaces, owning more pairs of shoes, and smoking. No protective factors were identified. In contrast, young runners had lower risk when running off-road, having a coach, using shoes with special features, and training on multiple surfaces. Risk increased with longer session duration and running on grass. Conclusion: These findings highlight the high prevalence of running participation limitations, particularly in master runners, and support the need for age-specific training and injury prevention strategies. Significance/Implications: Different age groups of runners may benefit from tailored risk mitigation and training modification strategies, as patterns of injury and contributing factors vary substantially between younger and master runners. Addressing these distinct needs has the potential to reduce injury-related disruptions and support long-term participation in running.
**Order of Authors:**	Rachel Berns
	Jo Armour Smith
	Natalia Sanchez
	Samantha Jeffcoat
	Susan Sigward
	Andrew Hooyman
	Shawn Farrokhi
**Additional Information:**	
**Question**	**Response**
**Financial Disclosure**<br><br>Enter a financial disclosure statement that describes the sources of funding for the work included in this submission. Review	The author(s) received no specific funding for this work.

Powered by Editorial Manager® and ProduXion Manager® from Aries Systems Corporation

the submission guidelines for detailed requirements. View published research articles from *PLOS ONE* for specific examples.<br><br>This statement is required for submission and **will appear in the published article** if the submission is accepted. Please make sure it is accurate.<br><br>**Funded studies**<br>Enter a statement with the following details:<br>• Initials of the authors who received each award<br>• Grant numbers awarded to each author<br>• The full name of each funder<br>• URL of each funder website<br>• Did the sponsors or funders play any role in the study design, data collection and analysis, decision to publish, or preparation of the manuscript?<br><br>Did you receive funding for this work?	
**Competing Interests**<br><br>Use the instructions below to enter a competing interest statement for this submission. On behalf of all authors, disclose any competing interests that could be perceived to bias this work—acknowledging all financial support and any other relevant financial or non-financial competing interests.<br><br>This statement is required for submission and **will appear in the published article** if the submission is accepted. Please make sure it is accurate and that any funding sources listed in your Funding Information later in the submission form are also declared in your Financial Disclosure statement.<br><br>View published research articles from *PLOS ONE* for specific examples.	The authors have declared that no competing interests exist.

**NO authors have competing interests**<br><br>Enter: *The authors have declared that no competing interests exist.*<br><br>**Authors with competing interests**<br><br>Enter competing interest details beginning with this statement:<br><br>*I have read the journal's policy and the authors of this manuscript have the following competing interests: [insert competing interests here]*<br><br>* typeset	
**Ethics Statement**<br><br>Enter an ethics statement for this submission. This statement is required if the study involved:<br><br>• Human participants<br>• Human specimens or tissue<br>• Vertebrate animals or cephalopods<br>• Vertebrate embryos or tissues<br>• Field research<br><br>Write "N/A" if the submission does not require an ethics statement.<br><br>General guidance is provided below. Consult the submission guidelines for detailed instructions. **Make sure that all information entered here is included in the Methods section of the manuscript.**	All participants provided written informed consent before completing any study surveys, and the study received approval from the Chapman University Institutional Review Board (IRB-24-138). The IRB-24-138 approval document has been provided for reference. No minors participated in this study.

**Format for specific study types**<br><br>**Human Subject Research (involving human participants and/or tissue)**<br>• Give the name of the institutional review board or ethics committee that approved the study<br>• Include the approval number and/or a statement indicating approval of this research<br>• Indicate the form of consent obtained (written/oral) or the reason that consent was not obtained (e.g. the data were analyzed anonymously)<br><br>**Animal Research (involving vertebrate animals, embryos or tissues)**<br>• Provide the name of the Institutional Animal Care and Use Committee (IACUC) or other relevant ethics board that reviewed the study protocol, and indicate whether they approved this research or granted a formal waiver of ethical approval<br>• Include an approval number if one was obtained<br>• If the study involved *non-human primates*, add *additional details* about animal welfare and steps taken to ameliorate suffering<br>• If anesthesia, euthanasia, or any kind of animal sacrifice is part of the study, include briefly which substances and/or methods were applied<br><br>**Field Research**<br><br>Include the following details if this study involves the collection of plant, animal, or other materials from a natural setting:<br>• Field permit number<br>• Name of the institution or relevant body that granted permission	
**Data Availability**<br><br>Authors are required to make all data underlying the findings described fully available, without restriction, and from the time of publication. PLOS allows rare exceptions to address legal and ethical concerns. See the PLOS Data Policy and FAQ for detailed information.	Yes - all data are fully available without restriction

Powered by Editorial Manager® and ProduXion Manager® from Aries Systems Corporation

A Data Availability Statement describing where the data can be found is required at submission. Your answers to this question constitute the Data Availability Statement and **will be published in the article**, if accepted.<br><br>**Important:** Stating 'data available on request from the author' is not sufficient. If your data are only available upon request, select 'No' for the first question and explain your exceptional situation in the text box.<br><br>Do the authors confirm that all data underlying the findings described in their manuscript are fully available without restriction?	
**Describe where the data may be found in full sentences. If you are copying our sample text, replace any instances of XXX with the appropriate details.**<br><br>• If the data are **held or will be held in a public repository**, include URLs, accession numbers or DOIs. If this information will only be available after acceptance, indicate this by ticking the box below. For example: *All XXX files are available from the XXX database (accession number(s) XXX, XXX.).*<br>• If the data are all contained **within the manuscript and/or Supporting Information files**, enter the following: *All relevant data are within the manuscript and its Supporting Information files.*<br>• If neither of these applies but you are able to provide **details of access elsewhere**, with or without limitations, please do so. For example:<br><br>*Data cannot be shared publicly because of [XXX]. Data are available from the XXX Institutional Data Access / Ethics Committee (contact via XXX) for researchers who meet the criteria for access to confidential data.*<br><br>*The data underlying the results presented in the study are available from (include the name of the third party*	All data used for this manuscript can be accessed at https://osf.io/ev8sc/overview?view_only=dbef68ab052746b998a5b1d3c710b1d9.

and contact information or URL).<br>• This text is appropriate if the data are owned by a third party and authors do not have permission to share the data.<br><br>* typeset	
Additional data availability information:	

Manuscript

Click here to access/download;Manuscript;Running Injury in Master Runners - PLOS One.docx

Variation in prevalence of participation limitation, injury location, and training habits between young and master runners: a cross-sectional study

Short Title: Running injury in master runners

Rachel Berns[1], Jo Armour Smith[2], Natalia Sanchez[2,3], Samantha Jeffcoat[2], Susan Sigward[1], Andrew Hooyman[2], Shawn Farrokhi[2]

[1]Division of Biokinesiology and Physical Therapy, University of Southern California, Los Angeles, CA, USA

[2]Department of Physical Therapy, Crean College of Health and Behavioral Sciences, Chapman University, Irvine, CA, USA

[3]Department of Electrical Engineering and Computer Science, Fowler School of Engineering, Chapman University, Orange, CA, USA

Corresponding Author E-mail: rberns@usc.edu (RB)

Abstract

Background/Objectives: Running is a popular form of physical activity, but its growing participation has been accompanied by high rates of injury that may limit ongoing engagement. This study explored differences in running participation limitation, injury location, and training characteristics between young (18–34 years) and master (>35 years) runners. Methods: A total of 207 runners (105 young, 102 master) completed an online survey assessing demographics, training habits, and injury-related participation limitations using the Oslo Sports Trauma Research Center Overuse Injury Questionnaire. Results: Master runners reported significantly higher rates of injury-related participation limitation than young runners (51% vs. 34%, $p<0.05$) and more upper extremity injuries ($p<0.01$). They also had longer running histories, higher weekly mileage, and longer training durations ($p<0.05$). Among master runners, risk factors for reduced participation included running on gravel/pebbled surfaces, owning more pairs of shoes, and smoking. No protective factors were identified. In contrast, young runners had lower risk when running off-road, having a coach, using shoes with special features, and training on multiple surfaces. Risk increased with longer session duration and running on grass. Conclusion: These findings highlight the high prevalence of running participation limitations, particularly in master runners, and support the need for age-specific training and injury prevention strategies. Significance/Implications: Different age groups of runners may benefit from tailored risk mitigation and training modification strategies, as patterns of injury and contributing factors vary substantially between younger and master runners. Addressing these distinct needs has the potential to reduce injury-related disruptions and support long-term participation in running.

Key Words: age-related differences, overuse, running injuries, training characteristics

Introduction

Running is one of the most widespread forms of physical activity globally, attracting individuals across a spectrum of participation, from casual recreational runners to competitive athletes [1]. Since 2020, the U.S. has seen a rise in recreational runners, along with increased running volume and frequency among experienced runners, partly due to the COVID-19 pandemic and the demand for exercise options that aligned with public health guidelines [2]. Much of the appeal to take up running as a form of exercise lies in its accessibility – running is low-entry level and requires minimal financial investment, making it a suitable activity for highly heterogeneous groups of people [3]. Given its broad appeal and health benefits, understanding the factors that influence running participation and associated injury risks is increasingly important.

The recent surge in running participation parallels an increase in running-related injuries (RRIs) [2]. Research has indicated that approximately 50% of runners will experience an RRI each year, highlighting the need for preventive measures [4]. A recent systematic review also reported that the overall prevalence of RRIs among runners is as high as 45%, with the knee, ankle, and lower leg diagnoses accounting for the highest proportion of RRI prevalence [5]. To this end, multiple factors have been identified to influence the rates of RRIs in runners. Demographic factors such as age, gender, and body weight have been shown to be associated with the presence of RRIs [6-9]. Training variables such as stretching before and after running [10], warm-up and cool-down routines, and running on hard surfaces can also influence both injury occurrence and severity [11-12]. Other factors, such as adherence to proper shoe usage, including the replacement of running shoes based on mileage rather than wear, can also influence the risk of RRIs [13]. Together, these findings underscore the multifactorial nature of RRIs and

the importance of investigating how modifiable and non-modifiable factors interact to influence injury risk and participation limitations.

Master runners, defined as runners aged 35 and older [14-15], are a distinct subset of the running population that are thought to engage in running for unique reasons beyond fitness alone. Previous research has corroborated that physical health benefits underscore running motivations for runners of all ages; however, master runners often place further emphasis on the social connections and emotional fulfillment derived from running [16]. These personal motivators may enhance the likelihood of sustained running participation over time, yet they are at odds with the higher injury rates experienced by older runners. Previous research has shown that master runners experience significantly higher overall injury rates compared to younger runners, with a greater prevalence of multiple and soft-tissue injuries, particularly in the calf, Achilles, and hamstrings [17]. These age-related differences in injury location and severity—along with differences in running habits such as higher weekly mileage, more frequent training sessions, and greater orthotic use—suggest that master runners may face distinct risks compared to their younger counterparts [17]. This highlights the need to examine how age influences running-related injury patterns, training behaviors, and participation limitations by directly comparing young and master runners.

The objectives of the current study were 1) to examine the differences in rates of running participation limitations and injury location between young and master runners, 2) to evaluate whether demographics, social factors, training characteristics and running habits are different between young and master runners, and 3) to determine what factors influence the risk of running participation limitation in young and master runners. We hypothesized that running participation limitation rates would be higher for master runners and that the location of their

RRIs, as well as their run training characteristics and habits, would be different from younger runners. In addition, we hypothesized that the factors associated with reduced or increased risk of running participation limitation would be different between young and master runners.

Methods

Participants

This study was conducted via an online survey completed by 207 adult runners from various regions across the United States from May 8th, 2024 to June 19th, 2024. Participants responded to recruitment flyers distributed through social media platforms, university campuses, emails to running clubs, and word-of-mouth referrals. To be eligible, individuals had to be at least 18 years old and currently engaged in a regular running program (defined as running 1–3 times per week for at least the three months prior to the survey), regardless of their level of experience. Given the broad inclusion criteria, all individuals who responded met eligibility requirements and completed the full survey; no participants were excluded or left the survey incomplete. Based on age, participants were categorized into two nearly equal groups: 105 young runners (aged 18–34 years) and 102 master runners (aged 35 years and older). All participants provided written informed consent before completing any study surveys, and the study received approval from the Chapman University Institutional Review Board (IRB-24-138).

Questionnaires and surveys

A previously developed survey [18] focusing on demographics and running characteristics and habits was converted into an electronic format and distributed via the protected web application REDCap (Research Electronic Data Capture) to all participants. The

survey comprised three sections: (a) questions related to participants' demographics and social behaviors, including age, sex, race, weight, height, education level, participation in other sports, and history of smoking or vaping; (b) questions regarding participants' running history and characteristics, such as the number of years they have been running, frequency of weekly running sessions, duration of training runs, mileage per training run, average weekly running mileage, preferred running races, use of special insoles, and number of running shoes in use; and (c) running training habits, including frequency of stretching, use of warm-up and cool-down routines, and the frequency of running sessions on various surfaces.

To assess limitations in running participation due to overuse injury and pain, the Oslo Sports Trauma Research Center Overuse Injury Questionnaire (OSTRC-O2) [19] was utilized. The OSTRC-O2 quantifies injury severity and its impact on participation in training or competition, modifications to training, performance limitations, and pain experienced during athletic activities over the last seven days. The OSTRC-O2 score is derived from four key questions, with a score of zero indicating no pain and full participation, while a score of 100 indicates severe pain and inability to participate.

The location of overuse injuries in runners was determined using a standardized body pain diagram, enabling participants to visually identify the specific area(s) of their injury. An RRI was defined as current musculoskeletal pain that restricted or halted running (in terms of distance, speed, duration, or training) for at least seven days, affected three consecutive scheduled training sessions, or necessitated consultation with a physician or other health professional [20]. Each runner was instructed to mark the body area(s) corresponding to their RRI(s). This visual approach is favored by both patients and clinicians, as it facilitates a more accurate and objective assessment of symptom distribution [21-22]. The marked areas were

subsequently categorized from 48 possible distinct body locations into eight mutually exclusive regions: 1) foot and ankle; 2) shin; 3) calf; 4) knee; 5) hip and thigh; 6) pelvis and low back; 7) upper back, ribs, and neck; and 8) shoulder, elbow, and hand.

Statistical analysis

All categorical variables were individually cross-tabulated with the two age categories (young/master) using chi-square tests of independence. Differences between groups for all continuous variables were compared using independent sample t-tests or Wilcoxon rank sum tests. To compare the rate of running participation limitation between young and master runners, participants were categorized based on their responses to the OSTRC-O2 questionnaire. Those reporting no RRIs, indicated by a score of zero on the OSTRC-O2, were classified as having "No participation restriction" over the previous seven days. In contrast, participants with any non-zero score, indicating reduced participation to complete inability to participate in running during the previous seven days, were classified as having "Minor to severe participation restriction." Group differences in participation restriction between young and master runners were analyzed using a chi-square test.

To identify and evaluate the variables with the most robust association between demographic characteristics, social factors, and training habits with participation restriction status, generalized LASSO (Least Absolute Shrinkage and Selection Operator) regression [23-24] was performed using the previously defined binary response categories ("No participation restriction" vs. "Minor to severe participation restriction") based on OSTRC-O2 scores. Two separate LASSO logistic regression models were constructed—one for young runners and one for master runners—to assess these associations within each cohort. The LASSO method applies an L1 penalty to the regression coefficients, promoting sparsity by shrinking less important

variables to zero, thereby identifying a minimal set of influential predictors. This approach is well-suited for handling correlated and high-dimensional predictors. Model tuning was conducted using stratified 10-fold cross-validation to select the optimal value of the regularization parameter (λ), that achieved the highest average area under the curve across all folds. All continuous predictors were standardized (mean = 0, standard deviation = 1) prior to modeling. By design, LASSO mitigates concerns of multicollinearity, allowing for robust variable selection even in the presence of interrelated predictors. These results reflect the variables selected by the LASSO regression model, which prioritizes parsimony and prediction over statistical significance testing. Because LASSO regression applies an L1 penalty that alters the distribution of coefficient estimates, traditional p-values are not directly available. Therefore, the interpretation of results focused on the strength and direction of associations based on odds ratios (OR) rather than statistical significance [24]. All analyses were performed using R version 4.4.0.

Results

Differences in participation limitation between young and master runners

A greater proportion of master runners (51%, 52 out of 102) reported OSTRC-O2 scores above zero compared to younger runners (34%, 36 out of 105), indicating some level of reduced or impaired participation in running over the previous seven days (p=0.04).

Differences in location of pain between young and master runners

Overall, the knee (27%-28%) and the foot/ankle (26%-27%) were the most frequently reported injury regions for both young and master cohorts, with no between-group differences (Fig 1). Of note, only a greater proportion of shoulder/arm/forearm injuries in master runners

was significantly different than younger runners (13% vs. 2%; p=0.008). Proportions across other body regions were not significantly different between age groups (Fig 1).

Fig 1. Bar graph representing the percentage of master and young runners reporting pain in specific body regions.

Differences in demographics and social habits between young and master runners

Table 1 presents the demographic and social characteristics of young and master runners. As expected, master runners were significantly older than younger runners (mean age: 43.7 vs. 25.5 years, p<0.001). Racial composition also differed significantly between the two cohorts (p=0.002), with a higher proportion of master runners identifying as White (58% vs. 48%) and Black or African American (28% vs. 14%), while a higher proportion of young runners identified as Asian (18% vs. 9%), or more than one race (8% vs. 2%). Educational attainment also differed significantly between groups (p<0.001), with younger runners more likely to have a high school diploma, whereas master runners more commonly held master's degrees or associate degrees. Although traditional smoking was more prevalent among master runners (12% vs. 4%), this difference did not reach statistical significance (p=0.06). However, e-cigarette use was significantly more common among young runners (36% vs. 22%, p=0.03). There was no significant difference between groups in terms of sex, body weight, height, or participation in other sports outside of running (p>0.05).

Table 1: Differences in demographics and social habits between Young and Master runners.

**Characteristic**	Younger, n=105[1]	Master, n=102[1]	p-value
**Age (years)**	25.5 (4.2)	43.7 (9.6)	**< 0.001[2]**
**Sex**			0.20[3]
Male	47 (45%)	56 (55%)	
Female	58 (55%)	46 (45%)	
**Body Weight (lbs)**	149.4 (26.8)	155.8 (28.9)	0.11[2]
**Height (inches)**	67.0 (3.4)	66.9 (4.2)	0.80[2]
**Race**			**0.002[3]**

Table 1: Differences in demographics and social habits between Young and Master runners.			
**Characteristic**	Younger, n=105[1]	Master, n=102[1]	p-value
White	50 (48%)	59 (58%)	
Black or African American	15 (14%)	29 (28%)	
Asian	19 (18%)	9 (9%)	
More than one race	8 (8%)	2 (2 %)	
Other	12 (11%)	3 (3%)	
**Ethnicity**			0.07[3]
Hispanic/Latino	24 (23%)	11 (11%)	
Non-Hispanic	80 (76%)	90 (88%)	
**Education Level**			**< 0.001[3]**
High School/GED	23 (22%)	2 (2.0%)	
Community College/Associate Degree	2 (1.9%)	8 (7.8%)	
B.S.	47 (45%)	48 (47%)	
Masters	13 (12%)	28 (27%)	
PhD/MD	20 (19%)	16 (16%)	
**Smoking Status (Yes)**	4 (3.8%)	12 (12%)	0.06[3]
**E-cigarette (Yes)**	38 (36%)	22 (22%)	**0.03[3]**
**Participated in Other Sports (Yes)**	54 (51%)	41 (40%)	0.14[3]

[1]Mean (SD); n (%)
[2] Two-sample t-test
[3] Chi-squared test

Differences in running history and training characteristics between young and master runners

Master runners had been running for more years (15.5 vs. 8.1; p<0.001), had longer average run training sessions (60.5 vs. 50.4 minutes; p=0.04), and ran a longer average distance per week (31.5 vs. 16.0 miles; p=0.04) compared to younger runners (Table 2). Master runners also owned more running shoes (3.9 vs. 2.8; p<0.001) and replaced their shoes more frequently within 6 months (p=0.04) than younger runners. Additionally, a higher proportion of master runners replaced their running shoes due to mileage versus wear and tear compared to younger runners (p=0.03), and a higher proportion of master runners had cushions or insoles in their running shoes compared to young runners (51% vs. 32%; p=0.03). In comparing the type of races each group participated in, a higher proportion of master runners ran in 10 km (35% vs.

20%; p=0.02) and 10 km to half-marathon (21% vs. 8%; p=0.01) races compared to younger runners.

Table 2: Differences in running history and training characteristics between Young and Master runners.

**Characteristic**	Younger, n=105[1]	Master, n=102[1]	p-value
**Years running**	8.1 (4.9)	15.5 (15.3)	**< 0.001[2]**
**Training sessions per week**	3.5 (1.4)	3.6 (2.9)	0.70[2]
**Average training session length (minutes)**	50.4 (33.6)	60.5 (38.3)	**0.04[2]**
**Average distance per week (miles)**	16.0 (14.8)	31.5 (70.9)	**0.04[2]**
**Average mile time**	10.0 (10.6)	10.2 (7.4)	> 0.90[2]
**Running coach? (Yes)**	42 (40%)	48 (47%)	0.40[3]
**Pairs of running Shoes**	2.8 (1.6)	3.9 (2.6)	**< 0.001[4]**
**Shoe replacement frequency**			**0.04[3]**
< 6 Months	11 (10%)	23 (23%)	
6 months – 1 Year	44 (42%)	45 (44%)	
1 Year – 1.5 Years	28 (27%)	23 (23%)	
1.5 years – 2 Years	19 (18%)	7 (6.9%)	
I don't know	3 (3%)	4 (4%)	
**Reason to replace shoes**			**0.03[3]**
Wear and Tear	86 (82%)	69 (68%)	
Mileage	19 (18%)	33 (32%)	
**Shoes with special features? (Yes)**	61 (58%)	61 (60%)	> 0.90[3]
**Shoe cushion or insole? Yes**	24 (32%)	33 (51%)	**0.03[3]**
**Do you wear orthotics? Yes**	15 (14%)	23 (23%)	0.20[3]
**What kind of race do you usually run?**			
Less than 10 km	54 (51%)	47 (46%)	0.50[3]
10 km	21 (20%)	36 (35%)	**0.02[3]**
>10 km - < ½ marathon	8 (8%)	21 (21%)	**0.01[3]**
½ Marathon	28 (27%)	37 (36%)	0.20[3]
Marathon	16 (15%)	17 (17%)	> 0.90[3]
Ultra marathons	3 (3%)	8 (8%)	0.20[3]
Don't run races	15 (14%)	6 (6%)	0.08[3]

[1]Mean (SD); n (%)
[2]Two-sample t-test
[3]Chi-squared test
[4]Wilcoxon rank sum test

Differences in training habits between young and master runners

Table 3 compares training habits between young and master runners. Overall, most training behaviors were similar between groups, with no significant differences in the frequency of stretching before or after training, warming up, cooling down, or training on common surfaces such as asphalt, off-road tracks, grass, cement, or treadmills (p>0.05). However, significant

group differences emerged for two training surface types. Young runners were more likely to train on gravel or pebbles compared to master runners (67% vs. 49%, p=0.02), while master runners reported training on "other surfaces" more frequently than younger runners (92% vs. 72%, p=0.01).

**Table 3. Differences in training habits between Young and Master runners.**			
**Characteristic**	Young, n=105[1]	Master, n=102[1]	p-value
**Do you stretch before training?**			0.11[2]
Never	64 (61%)	50 (49%)	
Sometimes/Always	41 (39%)	52 (51%)	
**Do you stretch after training?**			0.30[2]
Never	78 (74%)	68 (67%)	
Sometimes/Always	27 (26%)	34 (33%)	
**Do you warm up before training?**			> 0.90[2]
Never	49 (47%)	48 (47%)	
Sometimes/Always	56 (53%)	54 (53%)	
**Do you cool down after training?**			0.60[2]
Never	66 (63%)	59 (58%)	
Sometimes/Always	39 (37%)	43 (42%)	
**Do you train on asphalt?**			0.50[2]
Never/Rarely	83 (79%)	85 (83%)	
Sometimes/Often/Always	22 (21%)	17 (17%)	
**Do you train on off-road track?**			0.50[2]
Never/Rarely	65 (62%)	69 (68%)	
Sometimes/Often/Always	40 (38%)	33 (32%)	
**Do you train on grass?**			0.11[2]
Never/Rarely	42 (40%)	53 (52%)	
Sometimes/Often/Always	63 (60%)	49 (48%)	
**Do you train on gravel/pebbles?**			**0.02[2]**
Never/Rarely	35 (33%)	52 (51%)	
Sometimes/Often/Always	70 (67%)	50 (49%)	
**Do you train on cement?**			0.08[2]
Never/Rarely	75 (71%)	60 (59%)	
Sometimes/Often/Always	30 (29%)	42 (41%)	
**Do you train on a treadmill?**			> 0.9[2]
Never/Rarely	63 (60%)	63 (62%)	
Sometimes/Often/Always	42 (40%)	39 (38%)	
**Do you train on other surfaces?**			**0.01[2]**
Never/Rarely	21 (28%)	5 (8.5%)	
Sometimes/Often/Always	55 (72%)	54 (92%)	

[1]n (%); Mean (SD)
[2]Chi-squared test
[3]Wilcoxon rank sum test

Correlates of running participation limitation in young and master runners

Among young runners, several variables were associated with reduced odds of participation limitation. These included running on off-road tracks (OR: 0.68), having a running coach (OR: 0.70), wearing shoes with special features such as cushioning, stability, or motion control (OR: 0.75), and training on multiple surfaces (OR: 0.79). Slightly increased odds of participation limitation were associated with longer average training sessions (OR: 1.04) and running on grass (OR: 1.08), though these effects were modest. Among master runners, smoking (OR: 1.89), running on gravel or pebbled surfaces (OR: 1.10), and owning a greater number of pairs of running shoes (OR: 1.02) were associated with increased odds of participation limitation. No protective variables were identified for master runners.

Discussion

This study sought to explore how age influences running participation limitations, injury location, and training characteristics by comparing young and master runners. As hypothesized, master runners reported significantly higher rates of running participation limitation over the previous seven days as compared to younger runners. Although injury location did not differ substantially between groups for lower extremity joints, master runners were more likely to experience upper body injuries, specifically to their shoulders, arms, and forearms. Additionally, significant differences were observed in several demographic, social, and training-related characteristics between age groups. Master runners generally reported higher weekly mileage, longer run training sessions, and greater experience in the sport, alongside distinct equipment habits such as more frequent shoe replacement and use of insoles. While most training characteristics were similar across groups, surface preferences varied, with young runners more likely to train on gravel and master runners more likely to report use of other, unspecified surfaces. Finally, the factors associated with running participation limitation differed by age

group, supporting the hypothesis that the determinants of injury-related participation limitation are not uniform across the lifespan. Together, these findings underscore the importance of age-specific considerations when addressing injury prevention and training optimization strategies in the running population.

Differences in participation limitation between young and master runners

A significantly higher percentage of master runners in the current study reported having running participation limitations over the last seven days as compared to their younger counterparts. This finding aligns with one of the only previous studies comparing injury rates across similar age groups, which found master runners reported an injury rate of 49% over the previous year that affected their ability to train or race, which was significantly higher than younger runners at 45% [17]. These findings suggest that higher rates of RRIs can impair an individual's ability to maintain consistent running routines, as reflected in the greater participation limitations observed in the current study. This increase in participation limitations among master runners is concerning, particularly against the backdrop of the rising popularity of running. The documented link between running and heightened injury prevalence reflects a broader trend that necessitates targeted prevention strategies for aging runners. It is crucial that these findings inform future approaches to injury risk management, especially considering the differences in running habits and training characteristics in master runners.

Differences in location of pain between young and master runners

The injury patterns observed in this study align with prior research, with both young and master runners commonly reporting knee and foot/ankle injuries, reinforcing their status as the most frequently injured body regions across age groups [5, 17]. Interestingly, master runners in our study reported a higher proportion of upper extremity injuries. While this finding was

statistically significant, the low frequency of such injuries (only 13% of the total cohort) warrants cautious interpretation, as these cases may not reflect running-specific injuries but rather pre-existing conditions or non-running incidents that cause discomfort during running. Still, this observation highlights the need for further investigation into age-related biomechanical changes or training adaptations that could contribute to atypical injury sites and may inform more comprehensive approaches to injury prevention and rehabilitation in master runners.

Differences in demographics and social habits between young and master runners

The results of this study revealed significant differences between master and younger runners regarding racial identity, educational attainment, and e-cigarette use. The disparities in racial identity highlight potential predispositions to injury that may be influenced by cultural, social, or economic factors within different ethnic groups [25-26]. Notably, the higher education levels observed in master runners could be partially attributed to their older age, which may provide opportunities for advanced education. This may also reflect different access to resources or information regarding injury prevention. Conversely, the increased prevalence of e-cigarette use among younger runners introduces a new potential risk factor that warrants further investigation, given the known negative impact of vaping on respiratory health and athletic performance [27-28]. These findings emphasize the importance of considering demographic and social factors when evaluating injury risk and health behaviors among runners across different age groups.

Differences in running history and training characteristics between young and master runners

Despite their extensive running histories and commitment to longer training sessions, a higher percentage of master runners reported limitations in their participation due to RRIs, raising important questions about the long-term effects of sustained training on physical health. While years of consistent training enhance cardiovascular fitness and muscular endurance, the repetitive strain of increased mileage and intensity can lead to overuse injuries, as age-related degeneration, such as decreased muscle elasticity and reduced proprioception, heightens susceptibility to conditions like patellar tendinopathy and stress fractures [14, 29]. Additionally, the relationship between longer training sessions and fatigue compounds injury risk, as fatigue can impair biomechanical function and lead to altered running mechanics, further exacerbating the likelihood of new injuries [30-31]. Moreover, the cumulative training load experienced over time can lead to a threshold known as the "accumulated fatigue effect," wherein the body has not fully recovered from prior training loads, making a runner more vulnerable to injury during subsequent runs [32-33].

While the practice of owning and frequently replacing multiple pairs of running shoes reflects master runners' awareness of footwear's role in injury prevention [34] and may be related to their financial stability, it may also signify a direct response to these injuries. By rotating footwear and prioritizing mileage over wear and tear, master runners can reduce injury risks associated with overuse and maintain shoe performance [35]. Their use of cushioned insoles might further indicate adaptations made in response to past injuries or healthcare advice. Their continued participation in mid-distance races illustrates their dedication to the sport, yet cannot overshadow the limitations posed by injuries. Collectively, these behaviors - paired with their longer training histories, higher weekly mileage, and more frequent participation in 10 km to

half-marathon races - reflect both a greater training load and a proactive approach to injury mitigation in the face of age-related challenges.

Differences in training habits between young and master runners

Despite differences in age, training habits between young and master runners were largely similar, particularly regarding warm-up, cool-down, stretching routines, and use of common training surfaces such as asphalt, off-road tracks, and treadmills. However, surface preference differed in two notable ways. Young runners were more likely to train on gravel or pebbles, while master runners more frequently reported training on “other” surfaces. Gravel and pebble surfaces, known for their enhanced shock absorption and reduced impact on joints, can serve as a protective measure for younger runners against some overuse injuries. Research has shown that softer surfaces, like those composed of gravel, can diminish the repetitive stress on the knees and ankles, helping to prevent injuries such as patellar tendinopathy and stress fractures [12]. However, the instability and irregularity inherent in these surfaces can elevate the risk of acute injuries, such as falls and ankle sprains, necessitating careful attention to running technique and form. On the other hand, the preference to run on “other surfaces” observed in master runners may be due to individualized adaptations to minimize impact or accommodate existing injuries. These findings suggest that while foundational training behaviors are consistent across age groups, older runners may make surface-related adjustments to accommodate changing physical needs or injury histories.

Correlates of running participation limitation in young and master runners

The results of the regression analyses revealed distinct patterns in factors associated with running participation limitation between young and master runners, suggesting age-specific mechanisms underlying injury risk. Among young runners, protective associations with running

off-road, having a coach, wearing specialized footwear, and training on multiple surfaces may reflect the benefits of structured training environments, surface variability, and equipment tailored to individual biomechanics—factors that can mitigate overuse and repetitive strain injuries. Conversely, increased risk associated with longer training sessions and running on grass could indicate that exceeding individual training thresholds and running on uneven or softer terrain may predispose younger runners to overload or muscle injuries. For master runners, the absence of clear protective factors and the identification of risk associations with owning more running shoes, training on gravel or pebbles, and smoking may suggest a more complex injury profile. Rather than prevention, these behaviors could reflect post-injury adaptations or attempts to self-manage chronic injuries. For example, using multiple pairs of shoes and choosing specific surfaces may be reactive strategies to manage discomfort, while smoking has well-documented negative effects on tissue healing and musculoskeletal health [36-37]. Together, these findings point to the possibility that master runners experience more persistent or systemic barriers to injury recovery and participation, requiring targeted interventions that extend beyond surface-level training modifications.

Limitations

This study's limitations include several factors that may impact the robustness and generalizability of the findings. First, the reliance on self-reported data from a sample of 207 adult runners could introduce potential recall bias, as participants may misremember or underreport injury prevalence and training habits due to social desirability or lack of awareness. The study design, being cross-sectional, restricts the ability to draw causal inferences regarding the relationship between training behaviors and participation limitations. Longitudinal data collection would be necessary to observe changes over time and to identify trends in injury

incidence and participation limitations. A formal sample size calculation was not performed prior to study initiation. Instead, a minimum of 10 participants per predictor variable was targeted, following established guidelines for logistic regression analyses. With a final sample size of 207 runners, our study was able to include up to 20 predictor variables in the analysis of contributors to participation limitation. Based on the observed strength of associations in the results, the study is adequately powered to support the conclusions drawn regarding predictors of participation limitation in this population. This approach maximized feasibility within available resources while ensuring sufficient analytic capability for the main study objectives. Lastly, the study did not extensively explore the psychosocial factors influencing participation limitations, such as mental health, social support, and motivation, which could significantly contribute to understanding injury risk and running behaviors. This suggests a need for further research that integrates these dimensions to better inform injury prevention strategies among expanded runner demographics.

Conclusion

Understanding the rates and risk factors associated with running participation limitations is crucial for informing runners, coaches, and healthcare professionals about enhancing training practices while minimizing injury risks. As the popularity of running increases, it becomes essential to address injury rates through education and improved training protocols. This study offers insights, particularly regarding aging runners, and underscores the need for future research to adopt a multidimensional approach that examines both physiological and psychological aspects of running. Emphasizing education, tailored training practices, and the unique needs of master runners will help minimize the adverse effects of injury. Additionally, the observed relationships among demographic factors, height, shoe ownership, and age highlight the

importance of personalized training regimens that align with individual capabilities and injury histories. Encouraging runners to critically evaluate their training environments, especially concerning surface selection and shoe maintenance, can further mitigate injury risks. Successfully integrating these personalized elements into training strategies will be essential for fostering a sustainable and thriving running culture across all age groups.

Acknowledgements

We are grateful to all the individuals who participated in this study, and we acknowledge the valuable contribution of their time and personal experiences.

REFERENCES

1. Janssen M, Walravens R, Thibaut E, Scheerder J, Brombacher A, Vos S, et al. Understanding different types of recreational runners and how they use running-related technology. Int J Environ Res Public Health. 2020;17(7):2276 . doi: 10.3390/ijerph17072276.
2. DeJong AF, Fish PN, Hertel J. Running behaviors, motivations, and injury risk during the COVID-19 pandemic: A survey of 1147 runners. PLoS One. 2021;16(2):e0246300 . doi: 10.1371/journal.pone.0246300.
3. Cook S, Shaw J, Simpson P. Jography: Exploring Meanings, Experiences and Spatialities of Recreational Road-running. Mobilities. 2015;11(5):744-69 . doi: 10.1080/17450101.2015.1034455.
4. Dande V, Elahi J, Ganti L. Track and field-related lower-extremity musculoskeletal injuries: A retrospective study discussing injury analysis, stressors, and comprehensive management. Orthop Rev. 2024;16 . doi: 10.52965/001c.122542.
5. Kakouris N, Yener N, Fong DT. A systematic review of running-related musculoskeletal injuries in runners. J Sport Health Sci. 2021;10(5):513-22 . doi: 10.1016/j.jshs.2021.04.001.
6. Sanfilippo D, Beaudart C, Gaillard A, Bornheim S, Bruyere O, Kaux JF. What are the main risk factors for lower extremity running-related injuries? A retrospective survey based on 3669 respondents. Orthop J Sports Med. 2021;9(11):23259671211043444 . doi: 10.1177/23259671211043444.
7. Hollander K, Rahlf AL, Wilke J, Edler C, Steib S, Junge A, et al. Sex-specific differences in running injuries: A systematic review with meta-analysis and meta-regression. Sports Med. 2021;51(5):1011-39 . doi: 10.1007/s40279-020-01412-7.
8. van der Worp MP, ten Haaf DS, van Cingel R, de Wijer A, Nijhuis-van der Sanden MW, Staal JB. Injuries in runners; a systematic review on risk factors and sex differences. PLoS One. 2015;10(2):e0114937 . doi: 10.1371/journal.pone.0114937.
9. van Poppel D, van der Worp M, Slabbekoorn A, van den Heuvel SSP, van Middelkoop M, Koes BW, et al. Risk factors for overuse injuries in short- and long-distance running: A systematic review. J Sport Health Sci. 2021;10(1):14-28 . doi:

10.1016/j.jshs.2020.06.006.
10. Behm DG, Kay AD, Trajano GS, Alizadeh S, Blazevich AJ. Effects of stretching on injury risk reduction and balance. J Clin Exerc Physiol. 2021;10(3):106-16 . doi: 10.31189/2165-6193-10.3.106.
11. Ferro-Sánchez A, Martín-Castellanos A, de la Rubia A, García-Aliaga A, Hontoria-Galán M, Marquina M. An analysis of running impact on different surfaces for injury prevention. Int J Environ Res Public Health. 2023;20(14):6405 . doi: 10.3390/ijerph20146405.
12. Mitchell C, McDonnell S, Oganezova K, Mockler D, Fleming N. The effect of surface compliance on overground running biomechanics. A systematic review and meta-analysis. Sports Biomech. 2023;1–25 . doi: 10.1080/14763141.2023.2236058.
13. Malisoux L, Theisen D. Can the "appropriate" footwear prevent injury in leisure-time running? Evidence versus beliefs. J Athl Train. 2020;55(12):1215-23 . doi: 10.4085/1062-6050-523-19.
14. Willy RW, Paquette MR. The physiology and biomechanics of the master runner. Sports Med Arthrosc Rev. 2019;27(1):15-21 . doi: 10.1097/JSA.0000000000000212.
15. Ganse B, Degens H. Current insights in the age-related decline in sports performance of the older athlete. Int J Sports Med. 2021;42(10):879-88 . doi: 10.1055/a-1480-7730.
16. Ogles BM, Masters KS. Older vs. younger adult male marathon runners: Participative motives and training habits. J Sport Behav. 2000;23(2):130.
17. McKean KA, Manson NA, Stanish WD. Musculoskeletal injury in the masters runners. Clin J Sport Med. 2006;16(2):149-54 . doi: 10.1097/00042752-200603000-00011.
18. Hespanhol Junior LC, Costa LO, Carvalho AC, Lopes AD. A description of training characteristics and its association with previous musculoskeletal injuries in recreational runners: a cross-sectional study. Rev Bras Fisioter. 2012;16(1):46-53.
19. Clarsen B, Bahr R, Myklebust G, Andersson SH, Docking SI, Drew M, et al. Improved reporting of overuse injuries and health problems in sport: an update of the Oslo Sport Trauma Research Center questionnaires. Br J Sports Med. 2020;54(7):390-6 . doi: 10.1136/bjsports-2019-101337.
20. Yamato TP, Saragiotto BT, Lopes AD. A consensus definition of running-related injury in recreational runners: a modified Delphi approach. J Orthop Sports Phys Ther.

2015;45(5):375-80 . doi: 10.2519/jospt.2015.5741.
21. Southerst D, Côté P, Stupar M, Stern P, Mior S. The reliability of body pain diagrams in the quantitative measurement of pain distribution and location in patients with musculoskeletal pain: a systematic review. J Manipulative Physiol Ther. 2013;36(7):450-9 . doi: 10.1016/j.jmpt.2013.05.021.
22. Jang A, MacLean DL, Heer J. BodyDiagrams: Improving communication of pain symptoms through drawing. ACM Conference on Human Factors in Computing Systems (CHI), Toronto, Canada. 2014 . doi: 10.1145/2556288.2557223.
23. Ranstam J, Cook JA. LASSO regression. Br J Surg. 2018;105(10):1348 . doi: 10.1002/bjs.10895.
24. Tibshirani R. Regression shrinkage and selection via the lasso. J R Stat Soc Series B Stat Methodol. 1996;58(1):267-88 . doi: 10.1111/j.2517-6161.1996.tb02080.x.
25. Hill CN, Schmitt D, Reed W, Arent SM, Sands LP, Queen RM. Racial differences in running and landing measures associated with injury risk vary by sex. Sports Biomech. 2024;23(12):2738-56 . doi: 10.1080/14763141.2022.2056075.
26. Madsen C, Gabbe BJ, Holvik K, Alver K, Grøholt EK, Lund J, et al. Injury severity and increased socioeconomic differences: A population-based cohort study. Injury. 2022;53(6):1904-10 . doi: 10.1016/j.injury.2022.03.039.
27. Chaiton M, Pienkowski M, Musani I, Bondy SJ, Cohen JE, Dubray J, et al. Smoking, e-cigarettes and the effect on respiratory symptoms among a population sample of youth: Retrospective cohort study. Tob Induc Dis. 2023;21:08 . doi: 10.18332/tid/156839.
28. Hamann SL, Kungskulniti N, Charoenca N, Kasemsup V, Ruangkanchanasetr S, Jongkhajornpong P. Electronic cigarette harms: Aggregate evidence shows damage to biological systems. Int J Environ Res Public Health. 2023;20(19):6808 . doi: 10.3390/ijerph20196808.
29. Raiser SN, Schroeder AN, Lawley RJ, Tenforde AS. Bone health and the masters runner. PM R. 2024;16(4):363-73 . doi: 10.1002/pmrj.13175.
30. Winter S, Gordon S, Watt K. Effects of fatigue on kinematics and kinetics during overground running: a systematic review. J Sports Med Phys Fitness. 2017;57(6):887-99 . doi: 10.23736/S0022-4707.16.06339-8.
31. Zandbergen MA, Marotta L, Bulthuis R, Buurke JH, Veltink PH, Reenalda J. Effects of

level running-induced fatigue on running kinematics: A systematic review and meta-analysis. Gait Posture. 2023;99:60-75 . doi: 10.1016/j.gaitpost.2022.09.089.

32. Ristolainen L, Kettunen JA, Waller B, Heinonen A, Kujala UM. Training-related risk factors in the etiology of overuse injuries in endurance sports. J Sports Med Phys Fitness. 2014;54(1):78-87.
33. Jones CM, Griffiths PC, Mellalieu SD. Training load and fatigue marker associations with injury and illness: A systematic review of longitudinal studies. Sports Med. 2017;47(5):943-74 . doi: 10.1007/s40279-016-0619-5.
34. Sun X, Lam WK, Zhang X, Wang J, Fu W. Systematic review of the role of footwear constructions in running biomechanics: Implications for running-related injury and performance. J Sports Sci Med. 2020;19(1):20-37.
35. Malisoux L, Ramesh J, Mann R, Seil R, Urhausen A, Theisen D. Can parallel use of different running shoes decrease running-related injury risk?. Scand J Med Sci Sports. 2015;25(1):110-5 . doi: 10.1111/sms.12154.
36. Price TB, Krishnan-Sarin S, Rothman DL. Smoking impairs muscle recovery from exercise. Am J Physiol Endocrinol Metab. 2003;285(1):E116-22 . doi: 10.1152/ajpendo.00543.2002.
37. Chan SMH, Cerni C, Passey S, Seow HJ, Bernardo I, van der Poel C, et al. Cigarette smoking exacerbates skeletal muscle injury without compromising its regenerative capacity. Am J Respir Cell Mol Biol. 2020;62(2):217-30 . doi: 10.1165/rcmb.2019-01060.

Click here to access/download;Figure;Fig1.docx

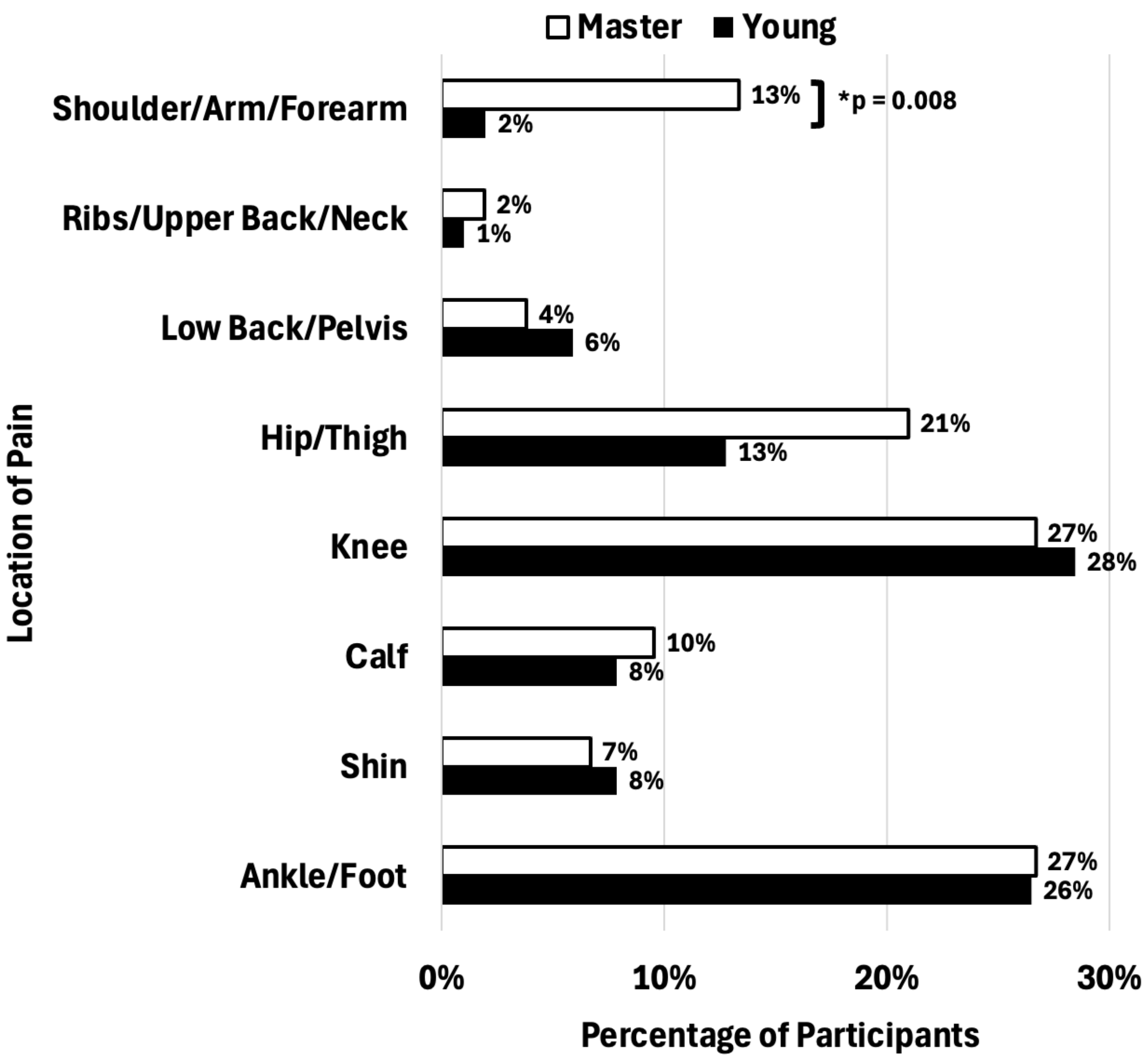

Fig 1. Bar graph representing the percentage of master and young runners reporting pain in specific body regions.
