# Supplementary material for: Variation in prevalence of participation limitation, injury location, and training habits between young and master runners: A cross-sectional study
Source: PLoS One. 2026 May 5;21(5):e0346973. doi: 10.1371/journal.pone.0346973 (PMC13143076; doi:10.1371/journal.pone.0346973)
Supplement: Attachment — Submitted filename: nnn.pdf [file pone.0346973.s002.pdf]

Overall Assessment

The manuscript presents a cross-sectional study examining differences in running-related participation limitations, injury locations, and training habits between young (18–34 years) and master (>35 years) runners. The topic is relevant to sports medicine and exercise physiology, addressing an understudied population of master runners amid growing recreational running participation. The study is well-motivated, with clear hypotheses and appropriate use of validated tools like the OSTRC-O2 questionnaire. However, limitations in sample size, lack of causal inference, and potential biases from self-reported data constrain the generalizability and depth of conclusions. The manuscript is suitable for publication in PLOS ONE with major revisions to enhance methodological rigor, statistical interpretation, and discussion of implications. Specific comments on each section follow.

Abstract

The abstract provides a clear overview of background, methods, results, and conclusions, adhering to PLOS ONE guidelines. Key findings on higher participation limitations in master runners (51% vs. 34%), upper extremity injuries, and age-specific risk factors are succinctly presented. Statistical significance is appropriately noted (e.g., $p<0.05$).

- The results list risk factors but could quantify odds ratios (ORs) for key associations (e.g., OR=1.89 for smoking in master runners) to provide more precise evidence.
- In the conclusion/implications, elaborate on practical applications, such as recommending coach-guided training for young runners.

Introduction:

The introduction effectively sets the context for rising running participation and associated injury risks, citing recent literature (e.g., post-COVID trends). It logically builds to the study's objectives and hypotheses, highlighting the unique motivations and risks for master runners. References are relevant and up-to-date.

- Strengthen the literature review by discussing potential mechanisms for age-related differences (e.g., biomechanical changes like reduced muscle elasticity [as later mentioned in discussion]) earlier to better justify hypotheses.

- Address gaps more explicitly: While prior studies on RRIs are cited, note the scarcity of comparative data on participation limitations specifically (e.g., beyond ref. [17]).

- The transition to objectives could include a brief rationale for the 35-year cutoff for "master" runners, referencing athletic federation standards.

Methods:

The methods describe participant recruitment, questionnaires, and statistical analyses adequately. The use of REDCap for data collection and LASSO regression for variable selection is innovative for handling multicollinearity. Ethical approvals and data availability are transparently reported.

- While the demographics/running habits survey is cited [18], specify any adaptations made for this study and report internal consistency (e.g., Cronbach's alpha) if applicable.

- Mention how the binary categorization ("No restriction" vs. "Minor to severe") was derived and justify the non-zero threshold, as milder scores might not always indicate clinically meaningful limitation.

- For injury location, describe inter-rater reliability in categorizing body diagram marks into regions, as subjective interpretation could introduce bias.

- Justify the choice of LASSO over other methods (e.g., stepwise regression) more explicitly. Report the lambda value selected via cross-validation and consider sensitivity analyses (e.g., varying thresholds). A power calculation retrospective to the sample size would bolster claims of adequacy (line 357–362).

- Discuss potential selection bias from social media and running clubs, which may favor more engaged or injured runners.

Results

Results are logically structured, with clear tables and a figure. Differences in participation limitations, injury locations, demographics, and training habits are well-presented, supported by appropriate tests (e.g., chi-square, t-tests). LASSO results highlight age-specific associations effectively.

- Tables 1–3: Add footnotes for abbreviations (e.g., SD) and ensure consistent formatting (e.g., p-values as "<0.001" vs. "0.002"). Consider combining tables for brevity if space allows.

- Figure 1: The bar graph is informative, but add error bars (e.g., 95% CI) and label p-values directly on bars for significant differences (e.g., shoulder/arm). Ensure accessibility (e.g., color contrast for print).
- Report ORs with 95% CIs to convey uncertainty, as p-values are unavailable. Discuss why certain variables (e.g., weekly mileage) were not selected by LASSO despite group differences.
- Clarify if multiple injuries per runner were accounted for in proportions (e.g., Fig 1 totals may exceed 100% if so).

Discussion

The discussion integrates findings with existing literature thoughtfully, addressing hypotheses and implications. Age-specific interpretations (e.g., reactive vs. preventive behaviors) add depth. Limitations are acknowledged candidly.

- Link upper extremity injuries to potential confounders like comorbidities or non-running activities, citing relevant studies.
- Provide actionable recommendations, such as periodized training for master runners to manage fatigue or footwear education programs. Discuss public health relevance, e.g., reducing healthcare burden from RRIs.
- Temper claims about causality (e.g., smoking's "negative effects" [line 344]—cite mechanisms specifically). Address conflicting evidence, e.g., if grass surfaces are protective in some studies but risky here.
- Integrate psychosocial factors mentioned in limitations earlier, as they could explain motivations for master runners [16].

Conclusion

The conclusion summarizes key insights and calls for multidimensional future research, aligning with PLOS ONE's emphasis on open science.

Strengthen by reiterating the novelty (e.g., age-specific risk profiles) and proposing specific study designs (e.g., prospective cohorts with objective measures like accelerometers).

- Avoid introducing new ideas (e.g., "relationships among demographic factors, height, shoe ownership" [line 379]—height was not discussed earlier; clarify or remove).

References

The reference list is comprehensive (37 citations), with a mix of recent (post-2020) and foundational works. DOIs are consistently provided.

- Ensure all citations are accurate (e.g., ref. [17] is from 2006—consider updating with more recent master runner studies if available).
- Check for completeness: Some sentences (e.g., line 292–294) cite mechanisms without specific references—add if possible.
-

Additional Comments

1. The threshold of 35 years for defining a "master runner" is commonly used in scientific literature but may be physiologically young, so highlight this limitation in the discussion and note that findings could differ for truly older individuals over 50 years.
2. Self-reported body diagrams for assessing injury location have low sensitivity and specificity, particularly in distinguishing soft tissues, joints, and nerves; add a cautionary statement in the limitations about diagnostic inaccuracies and recommend imaging or clinical evaluations in future studies.
3. The association between smoking and participation limitation in master runners (OR: 1.89) should be interpreted cautiously due to the non-significant group difference ($p=0.06$) and low number of smokers (12 in the master group).
4. The apparent contradiction where gravel/pebbled surfaces pose a risk for master runners (OR: 1.10) but not for young ones may indicate an age-surface interaction; analyze this in the discussion as potentially beneficial for younger bodies with higher flexibility but risky for older ones, and suggest testing age × surface interactions in future models.
5. Classifying all OSTRC-O2 scores greater than zero as participation limitation may include very mild pain without real functional impact; test a more meaningful threshold (e.g., ≥25 or ≥50) in sensitivity analyses or note in limitations that a zero threshold offers high sensitivity but low specificity.
6. Sampling via social media and running clubs may introduce self-selection bias favoring more engaged or injured individuals; describe this potential bias in the limitations and recommend future data collection from official marathon registries or national sports databases.
7. Owning more running shoes being linked to participation limitation in master runners may reflect a response to prior injuries rather than a cause; discuss this as

reverse causality in the discussion and advise controlling for previous injury history in future studies.

8. The phrase "running on gravel/pebbled surfaces" as a risk factor for master runners has an OR close to 1, indicating only slightly increased odds, which should be specified as such in the abstract for clarity.

Suggestion

The manuscript provides a valuable contribution by comparing participation limitations, injury patterns, and training characteristics between young and master recreational runners. However, to further strengthen the theoretical and practical grounding of the study—especially in the Introduction and Discussion sections—the authors are encouraged to incorporate recent, high-quality evidence focused specifically on recreational runners. In particular, the following studies directly address psychosocial, biomechanical, and multimodal intervention factors in this population and would enrich the manuscript's contextual framing and clinical implications:

- Adding mindfulness practice to exercise therapy for female recreational runners with patellofemoral pain: A randomized controlled trial
  – This trial highlights the role of non-biomechanical (e.g., psychological) factors in managing overuse injuries—a dimension currently underrepresented in your discussion, despite the use of the OSTRC-O2, which captures functional and pain-related limitations influenced by cognitive and emotional states.
- Predictors of Running-Related Injury Among Recreational Runners: A Prospective Cohort Study of the Role of Perfectionism, Mental Toughness, and Passion in Running
  – Given your finding that age-specific risk profiles exist, this study provides compelling evidence that personality and motivational traits independently predict injury risk in recreational runners. Integrating such psychosocial predictors would deepen the interpretation of why certain behaviors (e.g., higher mileage in master runners) may not be purely "training errors" but linked to deeper motivational drivers.
- Effect of arch support foot orthosis on pain severity in recreational runners with shin splint during running
  – Your results note differences in footwear use and surface training. This study offers direct experimental evidence on how biomechanical interventions (e.g., orthoses) modulate pain in common overuse conditions (e.g., shin splints), which aligns with your observations about shoe features and injury location.

- Effects of Integrating Lower-Leg Exercises Into a Multimodal Therapeutic Approach on Medial Tibial Stress Syndrome Management Among Recreational Runners: A Randomized Trial
  – This reference supports a more nuanced discussion of “reactive” vs. “preventive” behaviors in master runners (e.g., owning multiple shoes). It illustrates how targeted exercise, combined with other strategies, can alter injury trajectories—thus informing your recommendation for age-tailored prevention programs.
- Effectiveness of an online multi-component exercise-based injury prevention program on running-related injury risk in recreational runners: a randomized controlled trial
  – This study directly validates the feasibility and efficacy of scalable, age-inclusive prevention strategies—making it highly relevant to your conclusion that “age-specific training and injury prevention strategies” are needed. Citing this would strengthen the translational impact of your findings.

Incorporating these references would enhance the manuscript’s alignment with current evidence on multifactorial injury etiology and prevention in recreational runners, particularly by integrating psychological, biomechanical, and programmatic perspectives that complement your cross-sectional observations.
