# Supplementary material for: Variation in prevalence of participation limitation, injury location, and training habits between young and master runners: A cross-sectional study
Source: PLoS One. 2026 May 5;21(5):e0346973. doi: 10.1371/journal.pone.0346973 (PMC13143076; doi:10.1371/journal.pone.0346973)
Supplement: Attachment — Submitted filename: Response to Reviewers.docx [file pone.0346973.s003.docx]

March 03, 2026

Aynollah Naderi
Academic Editor
PLOS One

Dear Dr. Naderi,

Thank you for considering our manuscript entitled “Variation in prevalence of participation limitation, injury location, and training habits between young and master runners: a cross-sectional study.” We sincerely appreciate the careful and thorough review. We believe we have addressed all clarifying questions, and feel the paper is stronger as a result. Below are the authors’ point-by-point responses. In-text changes are highlighted in gray in the submitted revised manuscript for ease of review.

**Reviewer #1**

Thank you for submitting your manuscript entitled “Variation in prevalence of participation limitation, injury location, and training habits between young and master runners: a cross-sectional study” to PLOS ONE. Your cross-sectional investigation, based on survey data from 207 runners and employing the Oslo Sports Trauma Research Center Overuse Injury Questionnaire, provides valuable comparative insights into age-related differences in running-related participation limitations and associated factors. The findings regarding higher limitations in master runners (>35 years), increased upper extremity injuries, and distinct risk profiles contribute meaningfully to the literature on recreational running and support the call for age-specific prevention strategies.

The study has several strengths, including the use of a validated overuse injury tool, transparent reporting of ethics and data availability (via OSF), and clear presentation of age-specific LASSO regression results. However, several methodological and interpretive issues require attention to enhance scientific rigor and generalizability.

**Author’s Response: Thank you for your careful review and for highlighting the strengths and contributions of our manuscript. We appreciate the constructive feedback regarding methodological and interpretive considerations. We have now addressed all reviewer comments and revised the manuscript accordingly, which we believe has strengthened the study’s rigor and clarity, improved transparency around key analytic decisions, and more explicitly acknowledged factors that may affect generalizability.**

Background provided sufficient rationale for need for this research; purpose statement and hypotheses were clear. Methods was comprehensive and well organized. Results, discussion, and conclusion were consistent with the study's purpose; some brief additional statements are recommended for the initial discussion. Only very minor revisions are suggested. See pdf for specific comments.

**Author’s Response: We thank the reviewer for the constructive feedback and positive assessment of the manuscript. We appreciate the recommendation to include brief additional statements in the initial portion of the Discussion. We have reviewed the specific comments provided in the annotated PDF and revised the manuscript, adding clarifying language early in the Discussion to better contextualize the findings and strengthen the transition from the Results to the interpretation of clinical/research implications. All additional minor edits noted in the PDF have also been addressed.**

Line 72: the Achilles is part of the calf; suggest this; "...plantar flexor muscles, Achilles tendon..."

**Author’s Response: Thank you for this clarification. We revised the text (lines 80-83) to distinguish the calf/plantar flexor musculature from the Achilles tendon and to improve anatomical specificity.**

Line 235 (Discussion): prior to restating your purpose please also 1) briefly define the problem, ie, gap in the literature, and significance of the problem you attempted to address and 2) the need for this research and its clinical relevance.

**Author’s Response: Thank you for this suggestion. We revised the opening of the Discussion (lines 296 to 301) to briefly (1) restate the key gap in the literature and the significance of the problem, and (2) emphasize the need for this work and its clinical relevance before restating the study purpose.**

**Reviewer #2**

**Overall Assessment**

The manuscript presents a cross-sectional study examining differences in running-related participation limitations, injury locations, and training habits between young (18–34 years) and master (>35 years) runners. The topic is relevant to sports medicine and exercise physiology, addressing an understudied population of master runners amid growing recreational running participation. The study is well-motivated, with clear hypotheses and appropriate use of validated tools like the OSTRC-O2 questionnaire. However, limitations in sample size, lack of causal inference, and potential biases from self-reported data constrain the generalizability and depth of conclusions. The manuscript is suitable for publication in PLOS ONE with major revisions to enhance methodological rigor, statistical interpretation, and discussion of implications. Specific comments on each section follow.

**Author’s Response: Thank you for the detailed and constructive feedback. We appreciate the positive assessment of the study’s relevance, use of validated measures, and transparent reporting. We also appreciate the methodological and interpretive concerns raised. We have revised the manuscript accordingly and have addressed all subsequent comments provided by the reviewer in the sections that follow.**

**Abstract**

The abstract provides a clear overview of background, methods, results, and conclusions, adhering to PLOS ONE guidelines. Key findings on higher participation limitations in master runners (51% vs. 34%), upper extremity injuries, and age-specific risk factors are succinctly presented. Statistical significance is appropriately noted (e.g., p<0.05).

**Author’s Response:** **Thank you for your evaluation of the Abstract.**

The results list risk factors but could quantify odds ratios (ORs) for key associations (e.g., OR=1.89 for smoking in master runners) to provide more precise evidence.

**Author’s Response:** **Thank you for this suggestion. We have revised the Abstract to include the corresponding odds ratios for the age-specific factors associated with participation limitation to improve clarity and interpretability (lines 27-34).**

In the conclusion/implications, elaborate on practical applications, such as recommending coach-guided training for young runners.

**Author’s Response: Thank you for this suggestion. We revised the Conclusion/Significance – Implications to more explicitly describe practical applications based on our age-specific findings, including coach-guided training and training-surface/load considerations for young runners, and targeted risk mitigation (e.g., smoking status and surface exposure) for master runners (lines 37-42)**

**Introduction:**

The introduction effectively sets the context for rising running participation and associated injury risks, citing recent literature (e.g., post-COVID trends). It logically builds to the study's objectives and hypotheses, highlighting the unique motivations and risks for master runners. References are relevant and up-to-date.

**Author’s Response:** **Thank you for your feedback on the Introduction. We appreciate your assessment that it provides clear context, logically supports the study objectives and hypotheses, and includes relevant, current references. No changes were required in response to this comment.**

Strengthen the literature review by discussing potential mechanisms for age-related differences (e.g., biomechanical changes like reduced muscle elasticity [as later mentioned in discussion]) earlier to better justify hypotheses.

**Author’s Response: Thank you for this suggestion. We revised the Introduction (lines 80-86) to include a brief, mechanism-focused rationale describing age-related physiological and biomechanical changes (e.g., declines in aerobic capacity and muscle force-generating capacity and shifts in running mechanics/load distribution) that may contribute to differences in injury risk and participation limitation between young and master runners, thereby better supporting the study hypotheses.**

Address gaps more explicitly: While prior studies on RRIs are cited, note the scarcity of comparative data on participation limitations specifically (e.g., beyond ref. [17]).

**Author’s Response: Thank you for this comment. We revised the Introduction (lines 68-71) to more explicitly note that, although RRIs and their risk factors are well described, there is comparatively limited research directly comparing injury-related participation limitations between young and master runners, which motivated the current study.**

The transition to objectives could include a brief rationale for the 35-year cutoff for "master" runners, referencing athletic federation standards.

**Author’s Response: Thank you for this suggestion. We added a brief rationale (lines 74-76) in the Introduction noting that the ≥35-year threshold for “master” runners is consistent with commonly used competition-age classifications in track and field, including World Masters Athletics (WMA) and USA Track & Field (USATF), and we cited the relevant organizational sources.**

**Methods:**

The methods describe participant recruitment, questionnaires, and statistical analyses adequately. The use of REDCap for data collection and LASSO regression for variable selection is innovative for handling multicollinearity. Ethical approvals and data availability are transparently reported.

**Author’s Response:** **Thank you for your feedback on the Methods section. We appreciate your assessment that the recruitment procedures, measures, and statistical approach are adequately described, and that the use of REDCap and LASSO regression appropriately supports data quality and model development. We also appreciate your recognition of the transparent reporting of ethics approval and data availability. No changes were required in response to this comment.**

While the demographics/running habits survey is cited [18], specify any adaptations made for this study and report internal consistency (e.g., Cronbach's alpha) if applicable.

**Author’s Response: Thank you for this comment. We clarified in the Methods (lines 122-125, lines 133-139, and lines 142-144) that our demographics/running-habits survey was adapted from the instrument in Brazilian runners [18] and described the specific changes made to better fit our study aims and participant population, while maintaining the original content domains wherever possible. We also added a brief statement regarding reliability that because the demographics/training habits items are descriptive (not a single latent construct), internal consistency metrics such as Cronbach’s alpha are not appropriate. Additionally, the original study [18] did not report test–retest reliability, but the questionnaire has been used previously in runner cohorts to capture standard training and equipment variables, supporting its pragmatic use for cross-sectional characterization.**

Mention how the binary categorization ("No restriction" vs. "Minor to severe") was derived and justify the non-zero threshold, as milder scores might not always indicate clinically meaningful limitation.

**Author’s Response: Participants were dichotomized (OSTRC-O2 = 0 vs >0) because the OSTRC-O2 distribution in our sample was heavily right-skewed, with more than half of participants reporting a score of 0 (no injury/problems or participation limitations). In addition, the OSTRC-O2 scoring structure generates a more ordinal rather than interval scoring structure creating more discrete than continuous scores. Further, numeric changes in scores don’t necessarily represent a linear increase in injury (i.e., the item weighting creates non-linear score steps), which makes treating the total score as a continuous “severity” variable less desirable and increases the likelihood of violating key assumptions of univariate linear regression (e.g., normality, linearity, and homoscedasticity of residuals). We have clarified this rationale in the Methods (lines 148-153).**

For injury location, describe inter-rater reliability in categorizing body diagram marks into regions, as subjective interpretation could introduce bias.

**Author’s Response: Thank you for this comment. In our study, injury location was not coded via subjective interpretation of freehand body-diagram markings by the investigative team. Rather, participants completed an electronic body pain map with predefined selectable body locations and directly selected the location(s) corresponding to their injuries. The survey platform then automatically assigned each selection to one of the 48 discrete locations and, subsequently, the investigative team assigned each location to one of the eight mutually exclusive regions. Because no independent raters interpreted or re-coded participants’ markings, inter- and intra-rater reliability statistics are not applicable in this context. We have revised the Methods section (lines 164-168) to make this clearer by explicitly stating that participants selected injury locations from predetermined positions on the electronic body pain diagram and that region categorization followed from those selections.**

Justify the choice of LASSO over other methods (e.g., stepwise regression) more explicitly. Report the lambda value selected via cross-validation and consider sensitivity analyses (e.g., varying thresholds). A power calculation retrospective to the sample size would bolster claims of adequacy (lines 357–362).

**Author’s Response: We appreciate the reviewer’s thoughtful comments and have revised the manuscript accordingly. In the *Statistical Analysis* section, we now provide a clear rationale for selecting LASSO over stepwise regression, noting that LASSO offers greater stability in variable selection, handles correlated predictors more effectively, and reduces overfitting through cross-validated penalization (lines 208-213). We also added the requested reporting of the cross-validated penalty parameters in the results section (lines 278-279), stating that the optimal λ was 0.089 for the younger adult model and 0.119 for the master runner model. To address the recommendation for sensitivity testing, we incorporated a description of additional analyses in which each λ value was varied by ±20% to evaluate the robustness of the retained coefficients (lines 215-218); the *Results* section now reports that these coefficients remained stable across these perturbations (lines 288-294). Finally, a post hoc power analysis focused on variables selected by LASSO would be ill-advised, as selection under an L1 penalty alters coefficient distributions and renders conventional power calculations inappropriate for inference on selected predictors. Instead, we followed established adequacy guidelines for penalized/logistic modeling by targeting a parsimonious ratio of participants to candidate predictors (≈10 per predictor), which our final sample (N=207) met, supporting the suitability of our modeling strategy for the study’s primary objectives.**

Discuss potential selection bias from social media and running clubs, which may favor more engaged or injured runners.

**Author’s Response: We agree that our recruitment methods could introduce selection bias. Participants were recruited via social media, university campus flyers, emails to running clubs, and word-of-mouth referrals. These channels may preferentially reach runners who are more engaged with the running community, more frequent users of online platforms, or more motivated to participate in research—potentially including individuals currently experiencing pain or injury who may be more inclined to complete an injury-focused survey. Although we used multiple recruitment avenues and broad inclusion criteria to enhance reach, we acknowledge that the sample may not fully represent all U.S. runners (e.g., less-connected recreational runners). We have added this as a limitation (lines 474-480).**

**Results**

Results are logically structured, with clear tables and a figure. Differences in participation limitations, injury locations, demographics, and training habits are well-presented, supported by appropriate tests (e.g., chi-square, t-tests). LASSO results highlight age-specific associations effectively.

**Author’s Response: Thank you for your positive evaluation of the Results section. We appreciate the recognition that the results are logically structured and clearly presented, and that the statistical comparisons (chi-square and t-tests) and LASSO analyses effectively highlight age-specific associations. No changes were required in response to this comment.**

- Tables 1–3: Add footnotes for abbreviations (e.g., SD) and ensure consistent formatting (e.g., p-values as "<0.001" vs. "0.002"). Consider combining tables for brevity if space allows.

**Author’s Response: Thank you for the suggestion. We reviewed Tables 1–3 and addressed abbreviation clarity and formatting. All abbreviations in the tables have now been defined in table footnotes or replaced with the full terms to avoid ambiguity. We also standardized p-value reporting and now present exact p-values for all comparisons, except for extremely small p-values which are noted as 0.001 to ensure consistent formatting across tables. We also considered combining tables; however, we elected to retain Tables 1–3 as separate to preserve readability and maintain a clear separation between demographics, injury characteristics, and training variables (unless the journal requests further consolidation for space constraints). Our current results and methods section follow the data presentation depicted in our tables as currently constructed.**

- Figure 1: The bar graph is informative, but add error bars (e.g., 95% CI) and label p-values directly on bars for significant differences (e.g., shoulder/arm). Ensure accessibility (e.g., color contrast for print).

**Author’s Response: Thank you for your suggestions regarding Figure 1, which reports the observed percentages of participants in each age group who indicated pain in each body region. Error bars do not apply here because the bars are descriptive point proportions from the full study sample rather than estimates with sampling variability presented from a statistical model; adding error bars could imply precision/uncertainty that we are not estimating in this figure. To address the request to highlight significant differences, we labeled the p-value directly on the figure for the only region showing a significant between-group difference (shoulder/arm/forearm; p = 0.008). Finally, we used a high-contrast black-and-white format to improve accessibility (including for color-vision deficiencies) and to ensure clear reproduction in grayscale/print.**

- Report ORs with 95% CIs to convey uncertainty, as p-values are unavailable. Discuss why certain variables (e.g., weekly mileage) were not selected by LASSO despite group differences.

**Author’s Response: Thank you for this recommendation. Because our primary models used LASSO logistic regression, conventional p-values are not available and LASSO does not inherently provide valid 95% confidence intervals for the penalized coefficient estimates due to shrinkage and data-driven variable selection. Accordingly, we revised the manuscript (lines 290-294) to emphasize interpretation of the LASSO results in terms of the magnitude and direction of associations (ORs) rather than statistical significance testing, and we avoided presenting model-based CIs as if they were standard inferential quantities. We also expanded the explanation (lines 201-206) for why some variables (e.g., weekly mileage) were not selected by LASSO despite showing between-group differences in univariate analyses. As described in our Statistical Analysis section, LASSO applies an L1 penalty that shrinks less influential coefficients to zero, yielding a parsimonious subset of predictors. The model was tuned using stratified 10-fold cross-validation to select λ that maximized average AUC, meaning variables were retained based on incremental predictive value rather than statistical significance testing. Because LASSO is well-suited for correlated predictors and mitigates multicollinearity by selecting among interrelated variables, a factor such as weekly mileage may be excluded if its predictive information overlaps with other training variables or if it does not improve cross-validated model performance once other predictors are included. Consequently, variables can differ descriptively between groups yet be omitted by LASSO when they do not contribute uniquely to predicting participation restriction under the selected penalty. In addition, we fit separate LASSO models for young and master runners, so between-group differences (young vs master) are not evaluated within each model; instead, selection reflects which variables best predict participation limitation within each cohort.**

- Clarify if multiple injuries per runner were accounted for in proportions (e.g., Fig 1 totals may exceed 100% if so).

**Author’s Response: Participants were allowed to report multiple injury locations. Therefore, the proportions shown in Figure 1 represent the percentage of runners reporting pain in each body region (not mutually exclusive categories), and totals can exceed 100%. We have clarified this in both the Methods (lines 166-168) and the figure 1 legend.**

**Discussion**

The discussion integrates findings with existing literature thoughtfully, addressing hypotheses and implications. Age-specific interpretations (e.g., reactive vs. preventive behaviors) add depth. Limitations are acknowledged candidly.

**Author’s Response: Thank you for your thoughtful appraisal of the Discussion section. We appreciate your recognition that the discussion effectively integrates our findings with prior literature, addresses the study hypotheses and implications, and provides age-specific interpretations that add depth. We also appreciate your note that limitations were acknowledged candidly. No changes were required in response to this comment.**

- Link upper extremity injuries to potential confounders like comorbidities or non-running activities, citing relevant studies.

**Author’s Response: Thank you for this suggestion. We have now added a statement to the Discussion (lines 349-353) noting that upper extremity symptoms in master runners may be influenced by age-related comorbid musculoskeletal conditions (e.g., degenerative shoulder pathology) and/or non-running activities (e.g., occupational demands, resistance training, cycling, falls), and we incorporated supporting citations to relevant literature. This addition clarifies that the shoulder/arm/forearm findings should be interpreted cautiously and may not represent running-specific injury mechanisms.**

- Provide actionable recommendations, such as periodized training for master runners to manage fatigue or footwear education programs. Discuss public health relevance, e.g., reducing healthcare burden from RRIs.

**Author’s Response: Thank you for this suggestion. We have revised the Discussion to include more actionable, practice-oriented implications while remaining consistent with the cross-sectional nature of our data. Specifically, we added (1) a brief recommendation that clinicians/coaches consider emphasizing periodized training with planned recovery and symptom-guided load adjustment for master runners, given their higher training volumes and greater participation limitation (lines 396-399), and (2) a statement highlighting the public health relevance of reducing RRIs to support sustained physical activity and potentially reduce downstream healthcare utilization in the growing master-runner population (lines 336-339).**

- Temper claims about causality (e.g., smoking's "negative effects" [line 344]—cite mechanisms specifically). Address conflicting evidence, e.g., if grass surfaces are protective in some studies but risky here.

**Author’s Response: Thank you for this important comment. We revised the Discussion to further temper causal language and to clearly frame findings as associations, given the cross-sectional design of our study. Specifically, we softened statements that implied directionality (e.g., RRIs “impair” participation, lines 329-331) and revised statements about vaping (lines 365-368) and smoking (441-444) to describe plausible biological mechanisms (e.g., impaired tissue perfusion/collagen synthesis and delayed healing) rather than implying causation from our data. We also added a brief note (lines 428-431) acknowledging that evidence on running surface (grass) is mixed in the literature and that the observed association in our study may reflect contextual factors (e.g., uneven terrain, speed/intensity, exposure misclassification) and should be interpreted cautiously.**

- Integrate psychosocial factors mentioned in limitations earlier, as they could explain motivations for master runners [16].

**Author’s Response: Thank you for this suggestion. We have added text (lines 451-467) in the Discussion (Psychosocial Consideration and Multimodal Intervention Implications) to introduce these psychosocial considerations earlier and to link our findings to prior literature showing age-related differences in running motives (health/life meaning/affiliation vs. personal goal achievement) [16]. We also note that motivation in master runners is a distinct and important research area and that our team is currently conducting complementary work focused on these psychosocial contributors.**

**Conclusion**

The conclusion summarizes key insights and calls for multidimensional future research, aligning with PLOS ONE's emphasis on open science. Strengthen by reiterating the novelty (e.g., age-specific risk profiles) and proposing specific study designs (e.g., prospective cohorts with objective measures like accelerometers).

**Author’s Response: Thank you for this positive feedback. We appreciate your assessment that the Conclusion effectively summarizes the key insights and appropriately emphasizes the need for multidimensional future research, consistent with PLOS ONE’s scope and open-science priorities. We have revised the conclusion to better represent the findings of our study.**

- Avoid introducing new ideas (e.g., "relationships among demographic factors, height, shoe ownership" [line 379]—height was not discussed earlier; clarify or remove).

**Author’s Response: Thank you for noting this. We have removed this statement to ensure the Conclusion reflects only findings that were presented and interpreted earlier in the manuscript.**

**References**

The reference list is comprehensive (37 citations), with a mix of recent (post-2020) and foundational works. DOIs are consistently provided.

**Author’s Response: Thank you for your assessment of the reference list. We appreciate your note that the citations are comprehensive, appropriately balanced between recent and foundational works, and that DOIs are provided consistently. No changes were required in response to this comment.**

- Ensure all citations are accurate (e.g., ref. [17] is from 2006—consider updating with more recent master runner studies if available).

**Author’s Response: Thank you for this suggestion. We verified the accuracy of all citations, including ref. [17]. Although ref. [17] is from 2006, we did not identify a more recent study that provides a directly comparable young-versus-master runner injury prevalence estimate using similar definitions and reporting. We therefore retained this citation because it remains one of the few sources supporting the rationale for our age-stratified comparison and underscores the continued need for updated evidence—an important justification for the present study (lines 68-71).**

- Check for completeness: Some sentences (e.g., line 292–294) cite mechanisms without specific references—add if possible.

**Author’s Response: Thank you for this note. We have reviewed the revised manuscript and added references as needed.**

**Additional Comments**

1. The threshold of 35 years for defining a "master runner" is commonly used in scientific literature but may be physiologically young, so highlight this limitation in the discussion and note that findings could differ for truly older individuals over 50 years.

**Author’s Response: Thank you for this comment. We agree that the commonly used ≥35-year definition of “master runner” may be physiologically young and may not fully represent older runners. To address this, we added text early in the Discussion noting that while this cutoff aligns with standard research and competitive classifications, our findings may differ in truly older individuals (e.g., >50 years) and future studies should evaluate these relationships using more granular age categories (lines 303-306).**

2. Self-reported body diagrams for assessing injury location have low sensitivity and specificity, particularly in distinguishing soft tissues, joints, and nerves; add a cautionary statement in the limitations about diagnostic inaccuracies and recommend imaging or clinical evaluations in future studies.

**Author’s Response: Thank you for this important point. We agree that self-reported body pain diagrams may have limited diagnostic accuracy and can misclassify injury location/type (e.g., difficulty distinguishing soft-tissue, joint, or neural structures). We have therefore added a cautionary statement to the Limitations noting the potential for diagnostic inaccuracy and misclassification, and we now recommend that future studies incorporate clinical evaluation and/or imaging where feasible to improve diagnostic specificity. In addition, we clarified that the body pain map captured location only and did not assess pain intensity or pain quality (lines 488-494).**

3. The association between smoking and participation limitation in master runners (OR: 1.89) should be interpreted cautiously due to the non-significant group difference (p=0.06) and low number of smokers (12 in the master group).

**Author’s Response: Thank you for highlighting this. We agree that the observed association between smoking and participation limitation in master runners should be interpreted cautiously, given the small number of smokers in the master cohort (n=12) and the borderline between-group difference in smoking prevalence (p=0.06). We have revised the Discussion to explicitly note these limitations and to frame smoking as a hypothesis-generating correlate that requires confirmation in larger samples (lines 444-447).**

4. The apparent contradiction where gravel/pebbled surfaces pose a risk for master runners (OR: 1.10) but not for young ones may indicate an age-surface interaction; analyze this in the discussion as potentially beneficial for younger bodies with higher flexibility but risky for older ones, and suggest testing age × surface interactions in future models.

**Author’s Response: We appreciate this thoughtful comment. However, in our analyses, running on gravel/pebbles was associated with participation limitation in master runners, whereas no corresponding association was observed in young runners. Because we did not model a formal age × surface interaction term (and the young-runner estimate was not meaningfully different from null), we cannot confirm an interaction effect from these data, and we are concerned that discussing an age–surface interaction would be overly speculative given the study design and sample size. We therefore respectfully request not to include this interpretation in the revised manuscript.**

5. Classifying all OSTRC-O2 scores greater than zero as participation limitation may include very mild pain without real functional impact; test a more meaningful threshold (e.g., ≥25 or ≥50) in sensitivity analyses or note in limitations that a zero threshold offers high sensitivity but low specificity.

**Author’s Response: Thank you for this comment. As stated previously, we selected the binary categorization (OSTRC-O2 = 0 vs. >0) because the OSTRC-O2 distribution in our sample was strongly right-skewed with a large proportion of zeros, and because the OSTRC-O2 total score behaves more like an ordinal/non-linear composite than an interval-scaled severity measure; this rationale is now clarified in the Methods (lines 148-153).**

6. Sampling via social media and running clubs may introduce self-selection bias favoring more engaged or injured individuals; describe this potential bias in the limitations and recommend future data collection from official marathon registries or national sports databases.

**Author’s Response: Thank you for this suggestion. We have addressed this concern in the Limitations by explicitly noting that recruitment via social media, university campuses, emails to running clubs, and word-of-mouth may introduce self-selection bias that preferentially enrolls more engaged runners and/or individuals motivated to respond due to current symptoms or injury concerns, which could overestimate injury prevalence and participation limitation and reduce generalizability (lines 473-479).**

7. Owning more running shoes being linked to participation limitation in master runners may reflect a response to prior injuries rather than a cause; discuss this as reverse causality in the discussion and advise controlling for previous injury history in future studies.

**Author’s Response: Thank you for this important point. We agree that the association between owning more running shoes and participation limitation in master runners may reflect reverse causality (i.e., shoe acquisition/rotation as a response to prior or ongoing injury) rather than a causal risk factor. We have added concise language (lines 435-439) to the Discussion to clarify this and to recommend accounting for prior injury history in future work.**

8. The phrase "running on gravel/pebbled surfaces" as a risk factor for master runners has an OR close to 1, indicating only slightly increased odds, which should be specified as such in the abstract for clarity.

**Author’s Response: Thank you for this suggestion. To improve clarity, we have revised the Abstract to report the ORs for all associations, including gravel/pebble surfaces, so readers can interpret the magnitude of effects appropriately (lines 27-34).**

**Suggestion**

The manuscript provides a valuable contribution by comparing participation limitations, injury patterns, and training characteristics between young and master recreational runners. However, to further strengthen the theoretical and practical grounding of the study—especially in the Introduction and Discussion sections—the authors are encouraged to incorporate recent, high-quality evidence focused specifically on recreational runners. In particular, the following studies directly address psychosocial, biomechanical, and multimodal intervention factors in this population and would enrich the manuscript’s contextual framing and clinical implications:

- Adding mindfulness practice to exercise therapy for female recreational runners with patellofemoral pain: A randomized controlled trial – This trial highlights the role of non-biomechanical (e.g., psychological) factors in managing overuse injuries—a dimension currently underrepresented in your discussion, despite the use of the OSTRC-O2, which captures functional and pain-related limitations influenced by cognitive and emotional states.
- Predictors of Running-Related Injury Among Recreational Runners: A Prospective Cohort Study of the Role of Perfectionism, Mental Toughness, and Passion in Running – Given your finding that age-specific risk profiles exist, this study provides compelling evidence that personality and motivational traits independently predict injury risk in recreational runners. Integrating such psychosocial predictors would deepen the interpretation of why certain behaviors (e.g., higher mileage in master runners) may not be purely “training errors” but linked to deeper motivational drivers.
- Effect of arch support foot orthosis on pain severity in recreational runners with shin splint during running – Your results note differences in footwear use and surface training. This study offers direct experimental evidence on how biomechanical interventions (e.g., orthoses) modulate pain in common overuse conditions (e.g., shin splints), which aligns with your observations about shoe features and injury location.
- Effects of Integrating Lower-Leg Exercises Into a Multimodal Therapeutic Approach on Medial Tibial Stress Syndrome Management Among Recreational Runners: A Randomized Trial – This reference supports a more nuanced discussion of “reactive” vs. “preventive” behaviors in master runners (e.g., owning multiple shoes). It illustrates how targeted exercise, combined with other strategies, can alter injury trajectories—thus informing your recommendation for age-tailored prevention programs.
- Effectiveness of an online multi-component exercise-based injury prevention program on running-related injury risk in recreational runners: a randomized controlled trial – This study directly validates the feasibility and efficacy of scalable, age-inclusive prevention strategies—making it highly relevant to your conclusion that “age-specific training and injury prevention strategies” are needed. Citing this would strengthen the translational impact of your findings.

Incorporating these references would enhance the manuscript’s alignment with current evidence on multifactorial injury etiology and prevention in recreational runners, particularly by integrating psychological, biomechanical, and programmatic perspectives that complement your cross-sectional observations.

**Author’s Response: Thank you for these thoughtful suggestions and for recommending recent, high-quality studies in recreational runners. In response, we added a new Discussion subsection titled “Psychosocial considerations and multimodal intervention implications” (lines 451–467), which integrates this evidence by highlighting (1) the potential role of psychosocial traits and motivations (e.g., perfectionism/obsessive passion) in injury risk and participation decisions, (2) emerging support for mindfulness as an adjunct to exercise-based rehabilitation, and (3) evidence for multimodal and scalable prevention/rehabilitation approaches (e.g., multi-component exercise programs and condition-specific tools such as foot orthoses) that may inform age-tailored strategies for managing running-related pain and participation limitation.**
